# Case Report: Malignant Pheochromocytoma Without Hypertension Accompanied by Increment of Serum VEGF Level and Catecholamine Cardiomyopathy

**DOI:** 10.3389/fendo.2021.688536

**Published:** 2021-06-25

**Authors:** Hideaki Kaneto, Shinji Kamei, Fuminori Tatsumi, Masashi Shimoda, Tomohiko Kimura, Shuhei Nakanishi, Yoshiyuki Miyaji, Atsushi Nagai, Kohei Kaku, Tomoatsu Mune

**Affiliations:** ^1^ Department of Diabetes, Endocrinology and Metabolism, Kawasaki Medical School, Kurashiki, Japan; ^2^ Department of Diabetic Medicine, Kurashiki Central Hospital, Kurashiki, Japan; ^3^ Department of Urology, Kawasaki Medical School, Kurashiki, Japan

**Keywords:** malignant pheochromocytoma, noradrenaline, blood pressure, catecholamine cardiomyopathy, vascular endothelial growth factor

## Abstract

**Introduction:**

Pheochromocytoma is a catecholamine-producing tumor in the adrenal medulla and is often accompanied by hypertension, hyperglycemia, hypermetabolism, headache, and hyperhidrosis, and it is classified as benign and malignant pheochromocytoma. In addition, persistent hypertension is often observed in subjects with malignant pheochromocytoma.

**Case Presentation:**

A 52-year-old Japanese male was referred and hospitalized in our institution. He had a health check every year and no abnormalities had been pointed out. In addition, he had no past history of hypertension. In endocrinology markers, noradrenaline level was as high as 7,693 pg/ml, whereas adrenaline level was within normal range. Abdominal contrast-enhanced computed tomography revealed a 50-mm hyper-vascularized tumor with calcification in the right adrenal gland and multiple hyper-vascularized tumors in the liver. In ^131^I MIBG scintigraphy, there was high accumulation in the right adrenal gland and multiple accumulation in the liver and bone. In echocardiography, left ventricular ejection fraction was as low as 14.3%. In coronary angiography, however, there was no significant stenosis in the coronary arteries. Based on these findings, we finally diagnosed him as malignant pheochromocytoma accompanied by multiple liver and bone metastases and catecholamine cardiomyopathy. However, blood pressure was continuously within normal range without any anti-hypertensive drugs. Right adrenal tumor resection was performed together with left hepatic lobectomy and cholecystectomy. Furthermore, serum levels of vascular endothelial growth factor (VEGF) and parathyroid (PTH)-related protein were very high before the operation but they were markedly reduced after the operation.

**Conclusions:**

This is the first report showing the time course of serum VEGF level in a subject with malignant pheochromocytoma, clearly showing that malignant pheochromocytoma actually secreted VEGF. In addition, this case report clearly shows that we should bear in mind once again that malignant pheochromocytoma is not necessarily accompanied by hypertension.

## Introduction

Pheochromocytoma is a catecholamine-producing tumor in the adrenal medulla and is often accompanied by hypertension, hyperglycemia, hypermetabolism, headache, and hyperhidrosis. It is classified as benign and malignant pheochromocytoma, and histological grading of pheochromocytoma is usually evaluated with pheochromocytoma of the adrenal gland scaled score (PASS) (1–6). In malignant pheochromocytoma, distant metastasis and/or infiltration into surrounding tissues are observed. In addition, malignant pheochromocytoma usually secrete noradrenaline dominantly. Furthermore, PASS was ≥4 points in subjects with malignant pheochromocytoma. Regarding therapy for pheochromocytoma, the first choice is adrenal tumor resection. In case of unresectable tumors or metastases, the first choice is MIGB therapy. When it is difficult to perform MIBG therapy, chemotherapy using cyclophosphamide, vincristine, and dacarbazine is performed. Multi-kinase inhibitors such as sunitinib are also used at present. When pheochromocytoma is accompanied by hypertension, adrenergic α1 blocker is often used in order to reduce blood pressure. In addition, α1 blocker is often used before adrenal tumor resection in order to secure circulating plasma volume by blocking α-receptor stimulation and to avoid excessive drop of blood pressure after such operation.

It has been shown that vascular endothelial growth factor (VEGF) and its receptor are present in the adrenal gland in subjects with malignant pheochromocytoma and that the presence of them would be useful for differentiation between benign and malignant pheochromocytoma (7–9). It is also known that multi-kinase inhibitors such as sunitinib are effective especially in subjects with malignant pheochromocytoma producing VEGF (10). In addition, it has been reported that pheochromocytoma secrete parathyroid (PTH)-related protein (11, 12). Therefore, we measured serum levels of VEGF and PTH-related protein in this subject.

Here, we show a case who had malignant pheochromocytoma but did not have hypertension. In addition, such pheochromocytoma was accompanied by increment of serum levels of VEGF and PTH-related protein. Furthermore, drastic reduction of cardiac function was brought about by catecholamine cardiomyopathy in this subject. After right adrenal tumor resection, however, serum VEGF and PTH-related protein levels were normalized and heart failure was mitigated.

## Case Report

A 52-year-old Japanese male had cough, dyspnea, and lower leg edema for a couple of weeks. At first, he visited a primary physician but after then he was referred and hospitalized in our institution. This subject did not have any pheochromocytoma-specific complains such as headache, palpitation, and perspiration. He had a health check every year but no abnormalities had been pointed out. He had no past history of hypertension; his blood pressure had been within normal range without any anti-hypertensive drugs. This subject had no family history about diabetes mellitus, hypertension, dyslipidemia, or endocrine disorders except for stomach cancer in his father. On admission, his height, body weight, and body mass index were 178.0 cm, 66.0 kg, and 20.8 kg/m^2^. Blood pressure, heart rate, and body temperature were 110/78 mmHg, 118 beats/minute, and 37.0°C. **Table 1** shows clinical parameters on admission in this subject. Anemia and hypoalbuminemia were observed as follows: hemoglobin, 11.1g/dl; albumin, 2.6 g/dl. HbA1c was mildly higher, although fasting plasma glucose was within normal range. Moderate liver dysfunction was observed. In endocrinology markers, noradrenaline level was as high as 7,693 pg/ml (reference range: 100−450 pg/ml), whereas adrenaline level (40 pg/ml) was within normal range (reference range: 0−100 pg/ml). Dopamine level (29 pg/ml) was slightly higher compared to reference range (0−20 pg/ml). In urinalysis, similar results were obtained. Noradrenaline level in urine was as high as 2,946.6 µg/day (reference range: 48.6−168.4 µg/day), whereas adrenaline level (40 µg/day) was slightly higher compared to reference range (3.4−26.9 µg/day). Dopamine level in urine (1,175.9 µg/day) was slightly higher compared to reference range (365.0−961.5 µg/day). Brain natriuretic peptide (BNP) level was markedly increased up to 943.7 pg/ml (reference range: 0−18.4 pg/ml). It has been reported that VEGF and its receptor are present in adrenal tissues in subjects with malignant pheochromocytoma and that the presence of them would be useful for differentiation between benign and malignant pheochromocytoma (7–9). In addition, it was reported that multi-kinase inhibitors such as sunitinib are effective especially in subjects with malignant pheochromocytoma producing VEGF (10). VEGF level was also as high as 116 pg/ml (reference range: 0−38.3 pg/ml).

**Table 1:** Laboratory data on admission in this subject.

Peripheral blood		Endocrinology markers		Electrolytes	
RBC	403 × 10^4^/μl	ACTH	33.3 pg/ml	Sodium	139 mEq/L
Hemoglobin	11.1 g/dl	Cortisol	17.3 µg/dl	Potassium	4.8 mEq/L
Hematocrit	33.6%	DHEA-S	36 µg/dl	Chloride	105 mEq/L
WBC	5,460/µl	Renin	0.9 ng/ml/hr	Calcium	8.5 mg/dl
Platelet	22.1 × 10^4^/μl	Aldosterone	≤10.0 pg/ml	IP	3.3 mg/dl
**Blood biochemistry**		Adrenaline	40 pg/ml		
Total protein	6.6 g/dl	Noradrenaline	7,693 pg/ml	**Urinalysis**	
Albumin	2.6 g/dl	Dopamine	29 pg/ml	Total catecholamine	2,966.0 µg/day
Total bilirubin	0.3 mg/dl	TSH	3.95 µU/ml	Adrenaline	19.4 µg/day
AST	37 U/L	FT3	2.71 pg/ml	Noradrenaline	2,946.6 µg/day
ALT	60 U/L	FT4	0.82 ng/ml	Dopamine	1,175.9 µg/day
LDH	355 U/L	BNP	943.7 pg/ml	Total metanephrine	16.43 µg/day
*γ*-GTP	62 U/L	**Diabetes and lipid markers**		Metanephrine	0.23 µg/day
Creatinine	0.54 mg/dl	FPG	107 mg/dl	Normetanephrine	16.20 µg/day
BUN	11 mg/dl	HbA1c	7.0%		
Uric acid	6.1 mg/dl	LDL-cholesterol	73 mg/dl	**Other factors**	
Amylase	80 U/L	HDL-cholesterol	46 mg/dl	VEGF	116 pg/ml
CRP	2.93 mg/dl	Triglyceride	70 mg/dl	PTH-related protein	2.2 pmol/L

RBC, red blood cell; WBC, white blood cell; AST, aspartate aminotransferase; ALT, alanine aminotransferase; LDH, lactate dehydrogenase; γ-GTP, γ-glutamyl transpeptidase; BUN, blood urea nitrogen; CPR, C-reactive protein; ACTH, adrenocorticotropic hormone; DHEA-S, dehydroepiandrosterone sulfate; TSH, thyroid-stimulating hormone; FT3, free triiodothyronine; FT4, free thyroxine; BNP, brain natriuretic peptide; FPG, fasting plasma glucose; LDL, low density lipoprotein; HDL, high density lipoprotein; IP, inorganic phosphorus; VEGF, vascular endothelial growth factor; PTH, parathyroid hormone.

**Figure 1:**
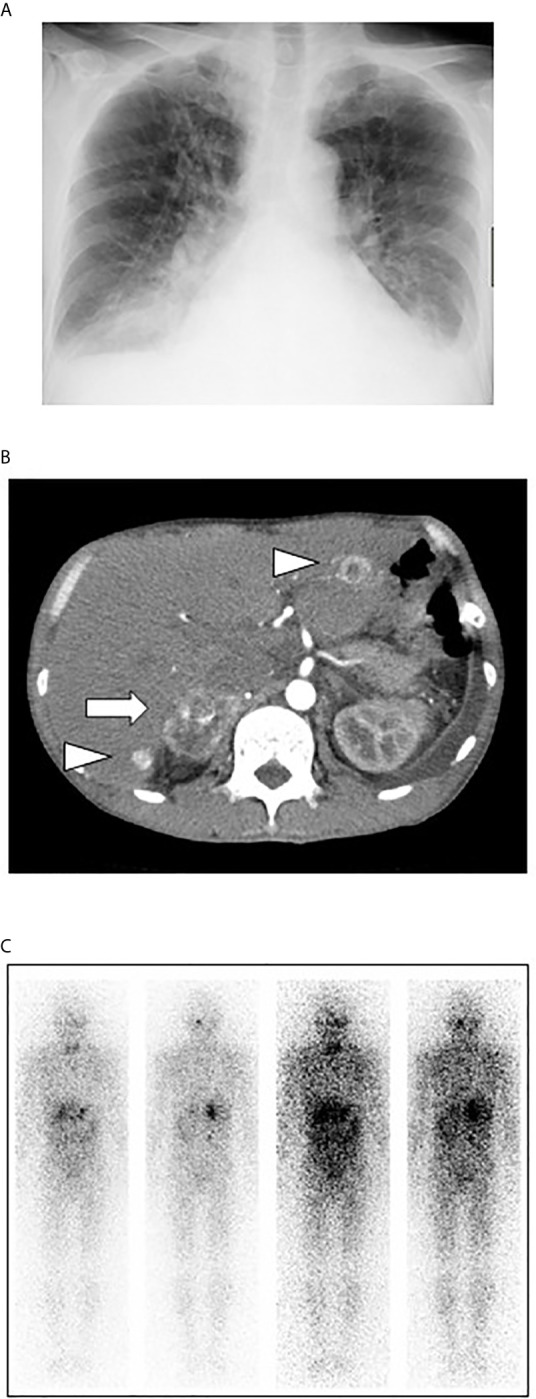
**(A)** In chest X-ray, permeability was reduced in bilateral lower lung field and butterfly shadow was observed. Cardio-thoracic ratio (CTR) was 59.8% and both costophrenic angles (CPA) were blunt. **(B)** Abdominal contrast-enhanced computed tomography revealed a 50-mm hyper-vascularized tumor with calcification in the right adrenal gland and multiple hyper-vascularized tumors in the liver. The right adrenal gland showed round shape and a mixture of high- and low-density area. In addition, since there was thin adipose tissue between the right adrenal gland and the kidney and inferior vena cava, there seemed to be no infiltration into surrounding organs. **(C)** In ^131^I MIBG scintigraphy, there was high accumulation in the right adrenal gland and multiple accumulation in the liver, and there was small hot spot in the sternum and right rib.

In chest X-ray, permeability was reduced in bilateral lower lung field and butterfly shadow was observed. Cardio-thoracic ratio (CTR) was 59.8% and both costophrenic angles (CPA) were blunt (**Figure 1A**). Abdominal contrast-enhanced computed tomography revealed a 50-mm hyper-vascularized tumor with calcification in the right adrenal gland and multiple hyper-vascularized tumors in the liver (**Figure 1B**). The right adrenal gland showed round shape and a mixture of high- and low-density area. In addition, since there was thin adipose tissue between the right adrenal gland and the kidney and inferior vena cava, there seemed to be no infiltration into surrounding organs. Similar findings were observed in the right adrenal gland and the liver in contrast-enhanced magnetic resonance imaging. In ^131^I MIBG scintigraphy, there was high accumulation in the right adrenal gland and multiple accumulation in the liver, and there was small hot spot in the sternum and right rib (**Figure 1C**). In echocardiography, left ventricular ejection fraction (LVEF) was as low as 14.3%, and left ventricular enlargement and diffuse wall motion abnormality were observed. In order to examine whether or not this subject had stenosis in the coronary arteries, coronary angiography was performed while blocking α-adrenergic pathway to secure circulating plasma volume. In coronary angiography, however, there was no significant stenosis in the coronary arteries. Based on various findings such as heart failure symptoms, increased catecholamine level, right adrenal tumor, accumulation in the adrenal gland, liver, and bone in ^131^I MIBG scintigraphy, we finally diagnosed him as malignant pheochromocytoma accompanied by multiple liver and bone metastases. Since there was no significant stenosis in the coronary arteries in coronary angiography, we thought that drastic reduction of cardiac function was brought about by catecholamine cardiomyopathy.

We started 20 mg of furosemide, 7.5 mg of tolvaptan for heart failure in this subject. Furthermore, we used 2,000 µg/day of carperitide for 1 week. BNP and CTR were gradually decreased by such treatment, and about 2 months later BNP and CTR were decreased to 262.8 pg/ml and 53.3%, respectively. LVEF was gradually increased by such treatment, and about 2 months later, LVEF was increased up to 23.7%. Although blood pressure was stable (approximately 100–120/60–80 mmHg), we started 4 mg of adrenergic α1 blocker doxazosin together with the above-mentioned furosemide or tolvaptan, and gradually increased its dose up to 16 mg before the operation in order to secure circulating plasma volume by blocking α1-receptor stimulation and to avoid excessive drop of blood pressure after the operation.

**Figure 2:**
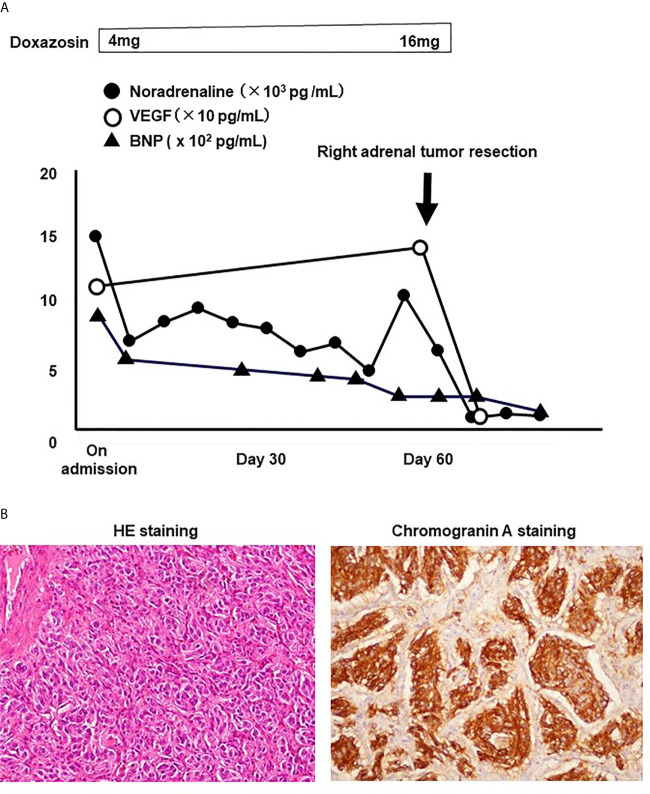
**(A)** Time course of noradrenaline, vascular endothelial growth factor (VEGF), and brain natriuretic peptide (BNP). Noradrenaline and VEGF levels were drastically decreased after right adrenal tumor resection, and BNP level was also gradually decreased before the operation. **(B)** In HE staining of the resected left hepatic lobe, there was small solid alveolar structure with a lot of relatively small cells and spindle-shaped nuclei were observed in some of such cells (left panel). In addition, in chromogranin A staining, many chromogranin A-producing cells were observed (right panel).

About 2 months later, right adrenal tumor resection was performed together with left hepatic lobectomy and cholecystectomy. As shown in **Figure 2A**, noradrenaline level was markedly decreased after the operation. VEGF level was as high as 144 pg/ml before the operation, but it was markedly decreased to <20 pg/ml after the operation (reference range: 0−38.3 pg/ml). These data clearly indicate that malignant pheochromocytoma in this subject actually secreted VEGF. In addition, parathyroid (PTH)-related protein level was also quite high before the operation (2.2 pmol/L), but it was markedly decreased to ≤1.0 pmol/L after the operation (reference range: 0−1.1 pmol/L), indicating that malignant pheochromocytoma in this subject secreted PTH-related protein as well. Calcium level and corrected calcium level were 8.5 and 9.9 mg/dl both of which were within reference range (8.5–10.4 mg/dl). In immunostaining of the resected adrenal gland, large alveolar structure, spindle-shaped cell pattern, vein invasion, and capsular invasion were observed. Thus, pheochromocytoma of adrenal gland scoring scale (PASS) was 7 points, indicating that pheochromocytoma in this subject was quite aggressive. In hematoxylin and eosin (HE) staining of the resected left hepatic lobe, there was small solid alveolar structure with a lot of relatively small cells and spindle-shaped nuclei were observed in some of such cells (**Figure 2B**). In addition, in chromogranin A staining, many chromogranin A-producing cells were observed. These data further strengthened the diagnosis that this subject had the metastasis of pheochromocytoma.

After the operation, radiation therapy (36Gy) was performed. In addition, chemotherapy was performed using cyclophosphamide, vincristine, and dacarbazine. Consequently, there was no recurrence for 2 years after the operation. Noradrenaline levels continued to be within normal range for 2 years. Furthermore, LVEF was increased up to around 40%, suggesting that that drastic reduction of cardiac function in this subject was brought about by catecholamine cardiomyopathy. It is noted, however, that MIBG therapy would have been more appropriate and have more favorable effects on this subject.

## Discussion

It has been reported that VEGF and its receptor are present in adrenal tissues in subjects with malignant pheochromocytoma and that the presence of them would be useful for differentiation between benign and malignant pheochromocytoma (7–9). In addition, it was reported that multi-kinase inhibitors such as sunitinib are effective especially in subjects with malignant pheochromocytoma producing VEGF (10). To the best of our knowledge, there was no report showing the time course of serum VEGF level in a subject with malignant pheochromocytoma. This is the first report showing the time course of serum VEGF level before and after the operation of pheochromocytoma. The data clearly demonstrate that malignant pheochromocytoma actually secreted VEGF in this subject. In addition, this report showed the alteration of PTH-related protein level before and after the operation. The data clearly demonstrate that malignant pheochromocytoma secreted PTH-related protein as well, although calcium level (8.5 mg/dl) and corrected calcium level (9.9 mg/dl) were within reference range (8.5−10.4 mg/dl).

In general, pheochromocytoma is one of endocrine disorders which often cause secondary hypertension. However, it was shown that pheochromocytoma was not necessarily accompanied by hypertension (13–16). Indeed, this subject had pheochromocytoma but blood pressure was within normal range without any anti-hypertensive drugs for a long period of time. In addition, characteristics of pheochromocytoma secreting noradrenaline dominantly are relatively high percentage of malignant pheochromocytoma and persistent hypertension. This subject had malignant pheochromocytoma secreting noradrenaline dominantly but blood pressure was continuously within normal range without any anti-hypertensive drugs. Therefore, we should bear in mind once again that malignant pheochromocytoma is not necessarily accompanied by hypertension. Also, we think that there are several possible reasons for the absence of hypertension. First, catecholamine may be converted to inactive metabolites inside tumor and then secreted into blood stream. Second, sensitivity of catecholamine receptor to catecholamine may be reduced by some reason. Third, some molecule may be secreted simultaneously and suppresses catecholamine action. Fourth, in case of this subject, significant decline of cardiac function due to catecholamine cardiomyopathy may be involved in the absence of hypertension. Fifth, since it is well known that VEGF facilitates nitric oxide production, leading to decrease of blood pressure, we think that VEGF secreted from pheochromocytoma may be associated with the absence of hypertension in this subject. It is noted, however, not all the patients with malignant pheochromocytoma have normal blood pressure and cardiac failure. In addition, this patient did not experience hypertension probably for many years from the early stage of pheochromocytoma when VEGF level was unlikely to be very high. Therefore, we assume that the elevated VEGF and PTH-related protein levels could be in context of cardiac failure as a compensatory mechanism.

In general, the prognosis of benign pheochromocytoma is relatively good, but that of malignant pheochromocytoma is quite poor. In many cases, malignant pheochromocytoma progressively worsens year by year, and 5-year survival rate is approximately 50% in subjects with malignant pheochromocytoma. Therefore, although fortunately there was no recurrence for 2 years after the operation in this subject, we think that we should follow up this subject very carefully for a long period of time.

There is a limitation in this report. First, we measured catecholamines which have a low half time and did not measure plasma levels of metanephrine and normetanephrine both of which are their metabolites. Second, we measured dopamine level but did not measure 3-metoxithyramine which are thought to be an important marker for malignant pheochromocytoma. Last but not least, MIBG therapy, but not radiation therapy and chemotherapy, would have been more appropriate and have more favorable effects on this subject.

Taken together, we experienced malignant pheochromocytoma without hypertension accompanied by increment of serum VEGF level and catecholamine cardiomyopathy. Therefore, it is likely that measurement of serum VEGF would be useful for diagnosis of malignant pheochromocytoma. In addition, we should bear in mind once again that malignant pheochromocytoma is not necessarily accompanied by hypertension.

## Author Contributions

HK, SK, FT, and TM researched data and/or wrote the manuscript. MS, TK, SN, YM, AN, and KK contributed to discussion. All authors contributed to the article and approved the submitted version.

## Conflict of Interest

The authors declare that the research was conducted in the absence of any commercial or financial relationships that could be construed as a potential conflict of interest.
